# Published Support for Wellness, Diversity, Equity, and Inclusion Among Internal Medicine Residency Program Websites

**DOI:** 10.7759/cureus.29328

**Published:** 2022-09-19

**Authors:** Kyle Storm, Gavin Kelly, Anita Kottapalli, Daniela Kaissieh, Victor Osio, Dani Zoorob

**Affiliations:** 1 Department of Obstetrics and Gynecology, The University of Toledo College of Medicine and Life Sciences, Toledo, USA

**Keywords:** inclusion and diversity, residency program website design, resident wellness, postgraduate training, personal characteristics/attitudes, admissions/selection/minority recruitment, continuing medical education

## Abstract

Introduction: The objective of this study was to review internal medicine residency program websites in the United States based on their published support for wellness, diversity, equity, and inclusion concepts. Inclusion of wellness, diversity, equity, and inclusion on program websites can serve as critical student benchmarks, and it may be paramount to optimize residency program websites accordingly.

Methods: This is a cross-sectional study of the websites of 597 internal medicine residency programs accredited by the Accreditation Council for Graduate Medical Education between March 25 and April 25, 2022. The websites were assessed based on 22 characteristics consisting of wellness verbiage, gender and underrepresented in medicine evaluation of faculty and residents, and diversity, equity, and inclusion-related semantics. Website photos were used to assess ethnic/sex representation. These attributes were devised by two sequentially set up focus groups consisting of 49 racially, ethnically, and gender-diverse medical students.

Results: A total of 579 internal medicine programs were reviewed. Only 239 (41%) had a dedicated page for resident wellness activities and efforts, while 134 (19%) had no mention of the concept throughout their web pages. Similarly, only 136 (23%) had a dedicated wellness officer, whether faculty or resident, who was focused on departmental interests. Gender diversity could be determined in 445 (77%) and 459 (79%) websites for faculty and residents, respectively. Underrepresented in medicine faculty and residents was noted in 293 (51%) and 393 (68%) of websites, respectively. A diversity, equity, and inclusion section was present in 172 (30%) of programs, with 93 (16%) having an assigned faculty or resident. Chairpersons or program directors stressed diversity, equity, and inclusion in up to 456 (79%) of the websites, with 181 (31%) having program mission statements or goals that include diversity, equity, and inclusion verbiage.

Conclusion: A deficit of various essential wellness, diversity, equity, and inclusion attributes persists across internal medicine residency websites. Residency programs would benefit from optimizing their websites to attract more diverse applicants.

## Introduction

Residency selection among medical students applying to internal medicine (IM) programs is a daunting task, as students must gauge the alignment of their values and interests against those of the programs they choose to apply to. There are currently 579 IM programs across the United States for rising medical students to select from, and the predominant method is obtaining information from program-specific websites [1]. This method of online navigation has become increasingly important in the residency selection process as the coronavirus disease 2019 pandemic has led programs to conduct applicant interviews in an exclusively virtual format. Resultingly, applicants draw their preliminary conclusions on program qualities from their virtual presence without the opportunity to explore select programs in person. Moreover, US medical graduates are increasingly applying to more programs, with an average of 73 applications during the 2021 cycle, more than twice as many as a decade ago [2]. The rising number of applications per student, along with decreased on-site visits to programs, places a heightened emphasis on residency programs' presence online.

Current literature describes many studies that target wellness and other diversity, equity, and inclusion (DEI) in residency programs and across various specialties [3-13]. However, few utilize one student-devised benchmarking, collection dependent entirely on student perceptions and efforts, and were comprehensive enough to assess both DEI and wellness.

Perceived goodness of fit and work/life balance rank among the most critical factors applicants consider when choosing a residency program [14-16]. Furthermore, existing workplace diversity and the overall climate for minority populations play a key role in program selection and long-term retention of residents of underrepresented minorities (underrepresented in medicine (URIM)) and female applicants [17]. The American Medical Association's adoption of national policies aimed at increasing DEI initiatives within programs has emphasized the importance of both diversity and wellness of residents. Similarly, Accreditation Council for Graduate Medical Education (ACGME) has amassed efforts to ensure the wellness of the resident physician workforce.

The objective of this study was to describe how IM programs across the United States focus on DEI and wellness based on a structured approach devised and implemented by prospective residency applicants.

## Materials and methods

This is an IM-focused secondary analysis of a cross-sectional qualitative study of ACGME-approved residency program websites across the United States between March 25 and April 25, 2022. The website assessment was based on a compilation of 22 attributes devised by two focus groups. The first focus group consisted of nine medical students who developed the criteria based on research and relevant online commentary. The second group comprised 40 voluntarily recruited students from a large Midwestern medical school. The second focus group piloted and refined the questions. The questions formed all focused on the presence of these attributes on program websites. The attributes assessed fit into three categories.

The first category is related to the concept of wellness. Wellness concepts assessed on program sites include mention of wellness in the department chairperson/program director's welcome letter or video, presence of a wellness page, presence of a wellness champion, and presence of group wellness activities among the residents. A wellness champion was defined as an appointed or elected individual (either faculty/resident) who was assigned to advocate for resident wellness. Group wellness activities were determined via descriptions of events or photos of events found on program websites.

The second category explored on program websites related to identity-related characteristics and concepts. This included any mention/emphasis of lesbian, gay, bisexual, transgender, queer/questioning, intersex, and asexual (LGBTQIA), use of inclusive pronouns, and gender diversity among faculty and residents. Mention of LGBTQIA was defined as any written or verbal reference to LGBTQIA initiatives, while emphasis was determined if an entire website section was dedicated to LGBTQIA causes. Gender diversity was determined by the medical student researcher's photo-based self-perceived identification of at least one member of a differing gender compared to the majority (a minimum of female and/or one male) within the faculty members and resident class.

Lastly, the concept of general equity and diversity-related characteristics was explored on program websites. Concepts assessed here include the presence of a dedicated DEI section, use of DEI verbiage in the chairperson/program director's letter or video, dedicated DEI officer or resident, racial and ethnic diversity in both residents and faculty, residents sharing of perspectives on DEI aspects of the program, resident involvement with underserved/lower socioeconomic communities, explicit incorporation of DEI in curriculum/curriculum objectives, DEI-focused research initiatives/projects displayed on websites, DEI verbiage in the mission statement or program goals, explicit mention of the holistic application review process, explicit mention of microaggression/implicit training bias for faculty/staff and residents, and explicit recognition of religious/cultural holidays with allocated resident time off. Racial and ethnic diversity was defined as the medical student researcher's self-perceived identification of at least one African American/Black, Hispanic/Latino, American Indian/Alaska Native, or Native Hawaiian/Pacific Islander faculty member and resident among their colleagues based on publicly accessible website photographic representation. Resident sharing of DEI perspectives was defined as direct quotes or videos of residents using DEI verbiage.

Thereafter, a cohort of racially, ethnically, and gender-diverse medical student researchers performed the website assessment based on the above-mentioned attributes to ensure representative benchmarking. Data were collected from the assessment and tabulated according to the respective DEI or wellness category. Analysis of the trends in diversity and wellness were computed using descriptive statistics.

A comprehensive list of accredited IM programs was obtained from the ACGME's website. To include programs in the assessment, each program had to be listed in the active ACGME listings, have a program identifiable online under the same name as in the list and utilizing the referenced ACGME program number, and have an accessible website with online content at the time of the study. Websites were visited three times at one-week intervals if not accessible during the initial search. Websites were excluded if their name correlated with more than one program, did not have full matriculation of all classes in each residency, and were not accessible upon searching.

## Results

A total of 579 residency programs (97% total, out of 597 programs listed) were analyzed based on the content of their websites. Up to 470 (81%) of the websites mentioned the concept of wellness, with 239 (41%) having a dedicated page. Only 136 (23%) had a wellness champion (resident or faculty), and 36 (6%) listed cultural competency as a component of their resident professional development events (Table 1).

**Table 1:** Presence of wellness characteristics on program websites

Concept		Programs	% of all programs
Mention of wellness in the chairperson or program director’s letter or video			
	Letter/page	106	18%
	Video	339	59%
	No mention	134	23%
Presence of a wellness-focused page			
	Wellness page with cultural competency efforts in the wellness events	36	6%
	Wellness page but no cultural competency	203	35%
	No wellness page but wellness is mentioned on the website	231	40%
	No wellness section	109	19%
Presence of a dedicated wellness champion (faculty/resident)			
	Wellness assigned faculty	58	10%
	Wellness assigned lead resident	78	13%
	No dedicated/assigned person	443	77%
Incorporation of group wellness activities amongst the residents			
	Yes	289	50%
	No	290	50%

Identity initiatives were limited to 125 (22%) mentioning the support of LGBTQIA personnel and 12 (2%) utilizing inclusive pronouns on their websites. Gender diversity was clearly noted in 445 (77%) of websites in relation to faculty and 459 (79%) in residents. A total of 105 (18%) and 113 (20%) program websites did not clearly determine faculty and resident gender diversity respectfully (Table 2). The residency websites also suggested that racial and ethnic diversity was more likely to be among resident members (393, 68%) compared to faculty (293, 51%) (Table 3).

**Table 2:** Presence of identity-related characteristics on program website LGBTQIA = lesbian, gay, bisexual, transgender, queer/questioning, intersex, asexual, and agender.

Concept		Programs	% of all programs
Mention of LGBTQIA			
	Mention	103	18%
	Emphasize	22	4%
	No mention/verbiage	453	78%
Presence of inclusive pronouns			
	Yes	12	2%
	No	567	98%
Faculty gender diversity			
	Cannot be determined	105	18%
	Yes (% based on all reviewed websites)	445	77%
	No (% based on all reviewed websites)	29	5%
Resident gender diversity			
	Cannot be determined	113	20%
	Yes (% based on all reviewed websites)	459	79%
	No (% based on all reviewed websites)	7	1%

**Table 3:** Presence of general equity and diversity-related characteristics on program website DEI = diversity, equity, and inclusion; URIM = underrepresented in medicine.

Concept		Programs	% of all programs
A website with a dedicated DEI section			
	Yes	172	30%
	No	405	70%
DEI verbiage in the chairperson or program director’s letter or video			
	Letter/page	129	22%
	Video	327	56%
	No mention	123	21%
Dedicated DEI oﬃcer or resident			
	Yes	93	16%
	No	486	84%
Diversity in faculty (URIMs)			
	Cannot be determined	124	21%
	Yes (% based on all reviewed websites)	293	51%
	No (% based on all reviewed websites)	162	28%
Diversity in residents (URIMs)			
	Cannot be determined	128	22%
	Yes (% based on all reviewed websites)	393	68%
	No (% based on all reviewed websites)	58	10%
Resident sharing of perspectives on the DEI aspect of the program			
	Yes	67	12%
	No	511	88%
Resident involvement with underserved/lower socioeconomic communities			
	Yes	253	44%
	No	326	56%
Explicit incorporation of DEI in curriculum/curriculum objectives			
	Yes	99	17%
	No	479	83%
DEI-focused research initiatives/projects displayed on the website			
	Yes (% based on websites with available listed research)	57	21%
	No (% based on websites with available listed research)	221	80%
	No research listed (% based on all reviewed websites)	301	52%
	Yes (% based on all reviewed websites)	57	10%
	No (% based on all reviewed websites)	221	38%
DEI verbiage in the mission statement or program goals			
	Yes	181	31%
	No	398	69%
Explicit mention of the holistic application review process			
	Yes	64	11%
	No	515	89%
Explicit mention of microaggression/implicit bias training for residents			
	Yes	50	9%
	No	529	91%
Explicit mention of microaggression/implicit bias training for faculty/staff			
	Yes	48	8%
	No	531	92%
Recognition of religious/cultural holidays with allocated resident time off			
	Yes	14	2%
	No	564	98%

Residency-related DEI focus was noted in 172 (30%) of websites through dedicated web pages and 456 (79%) through leadership verbiage, whether in video or written format. DEI verbiage was present in 181 (31%) of mission statements or program goals. Additionally, 99 (17%) of programs explicitly listed the incorporation of DEI in their curriculum or its objectives, and 93 (16%) appointed dedicated DEI champions to bolster departmental efforts (Table 3).

## Discussion

IM residency programs adequately emphasize wellness and routinely demonstrate gender diversity amongst faculty and residents on their websites; however, any mention of LGBTQIA is minimal on program websites. There is also room for developing more comprehensive DEI sections that allow for displaying DEI initiatives included in the curriculum.

With increasing rates of physician burnout and generational shifts in interests, emphasis on wellness in medicine has become vital, especially when selecting a residency program [14]. Residency-integrated wellness programs are shown to significantly improve resident quality-of-life scores and reduce anxiety and fatigue [3,4]. Based on our study findings, the importance of this concept is evident through having the majority of programs (470, 81%) demonstrate their support for the trainees’ needs. Similarly, up to 289 (50%) of residency websites reported on group wellness activities, with research suggesting that such incorporation of efforts into resident activities and program curricula was positively perceived by applicants [5-10]. The websites surveyed stressed cultural competency in only 36 (6%), and less than a quarter had identified faculty or residents assigned such a focus. Both cultural competency training and champion assignment may emphasize the departmental investment in well-being and offer the opportunity for resident engagement in the efforts.

Most IM program websites have images readily available of faculty and residents for applicants to view, demonstrating a clear presence of gender diversity. However, some of the accredited programs lacked clear evidence of gender diversity, resulting in an inability to assess gender distribution, potentially impacting applicant consideration and ranking, especially in females [18]. The presence of photographs on a program's website is also essential for applicants who are minorities. URIM applicants have rated faculty and house staff diversity as necessary when deciding what residency program to join compared to non-minority applicant colleagues [18]. Additionally, feelings of isolation are more prevalent in minority residents, depending on the level of social support and mentorship in residency programs. URIM residents have noted that having faculty members representing them and emphasizing their background was paramount and that diversity was vital in medical education [19]. Thus, having a website that emphasizes diversity, at least by using staff photographs, could be instrumental in recruiting a more racially and ethnically diverse class that more accurately reflects the patient population distribution. Additionally, program sites could make URIM applicants feel more welcomed by dedicating a page to DEI and assigning DEI champions - two cost-effective measures that could improve recruitment of a diverse residency class.

Similarly, LGBTQIA healthcare professionals are another group underrepresented in training [11]. As no standardized methodology exists to engage the LGBTQIA community, emphasizing support on the website may have similar effects on recruitment as gender diversity. However, IM residency program websites appeared to support in 125 (22%) of cases, with only 12 (2%) displaying inclusive pronouns. Although not verified as disinterest, this lack of evident support has significantly burdened the applicants, especially with the switch to virtual interviews [12,13]. The lack of inclusive pronouns has also been a deterrent to certain applicants. Efforts to support the community have varied, with one residency program initiating a focused effort in which LGBTQIA residents met specifically with LGBTQIA applicants during interviews, which raised the average of three residents in the program prior to seven the following year [11]. However, recruitment-related publications have focused on having information ready on the website as well as during meetings to help offer interest without prompting by the applicant [13].

This study has several limitations, including the lack of inter-rater reliability (formal validation of the tool used) when assessing the websites and the reliance on website content that may be outdated and/or limited by standardized institutional website criteria. Similarly, the students' perceptions of racial and ethnic representation are subjective as they rely on photographs, and this may not reflect the actual program's diversity. Additionally, considering one person per group as sufficiently representative of gender or racial/ethnic diversity is an understatement, as representation needs to be aligned with national or community-based percentages. Strengths of the study include the comprehensive analysis platform used that was devised by a medical student. An additional strength is the data collection being performed by students interested in the specialty and intending to apply within the year.

## Conclusions

Residency program websites for IM need to emphasize wellness and DEI further. Websites can improve by having dedicated wellness and DEI pages. Support for LGBTQIA healthcare professionals could be better represented on websites. Additional studies are needed to explore how each attribute affects a medical student's decision-making. Improvements may help attract a diverse applicant pool while ensuring the resilience of the trainees.
